# Effects of household chaos and parental responsiveness on child executive functions: a novel, multi-method approach

**DOI:** 10.1186/s40359-021-00651-1

**Published:** 2021-09-22

**Authors:** Krysta Andrews, James R. Dunn, Heather Prime, Eric Duku, Leslie Atkinson, Ashwini Tiwari, Andrea Gonzalez

**Affiliations:** 1 Department of Psychiatry and Behavioural Neurosciences, McMaster University, 1280 Main St. W, MIP 201A, Hamilton, ON L8S 4K1 Canada; 2 Department of Health, Aging, and Society, McMaster University, 1280 Main St. W, Hamilton, ON L8S 4K1 Canada; 3 Department of Psychology, York University, 4700 Keele St., Toronto, ON M3J 1P3 Canada; 4 Offord Centre for Child Studies, McMaster University, 1280 Main St. W, Hamilton, ON L8S 4K1 Canada; 5 Department of Psychology, Ryerson University, 350 Victoria St., Toronto, ON M5B 2K3 Canada; 6 Institute of Public and Preventive Health, Augusta University, 1120 15th St., Augusta, GA 30912 USA

**Keywords:** Household chaos, Child, Executive functions, Parental responsiveness

## Abstract

**Background:**

Executive functions can be adversely affected by contextual risks in the home environment including chaos and parenting challenges. Furthermore, household chaos negatively influences parenting practices. Few studies, however, have examined the role of parenting in the association between household chaos and child executive functions.

**Methods:**

Using a sample of 128 school-aged children (mean = 61.9 months, SD = 2.0, range 58–68 months) and their mothers, the present study examined direct and indirect effects (via parental responsiveness) of household chaos on child executive functioning. Multi-measures were used including performance-based assessments, behavioural observations, questionnaires, and video-home tours.

**Results:**

Household chaos had both a direct effect on child executive functions (β = − .31, 95% CI [− .58, − .04]) and an indirect effect (β = − .05, 95% [− .13, − .01]) via parental responsiveness. Further, the indirect effect was only significant for household instability.

**Conclusion:**

These findings indicate that parental responsiveness may be compromised by household chaos, with implications for the executive functions of school-aged children. Preventative strategies are needed to improve the stability in the home and strengthen parenting practices.

## Background

The development of executive functions in early childhood plays an important role in shaping the overall functioning of the child. Executive functions describe neurocognitive processes that modulate one’s thoughts, emotions and actions in goal directed behaviours [1]. Inhibition, working memory and cognitive flexibility are commonly explored as core executive functions in early childhood research. Inhibition refers to the ability to suppress automatic or dominant responses via control over attention, thoughts, emotions, and actions [1]. Cognitive flexibility describes switching between modes of mental operations, tasks, or cognitive rules [2].Working memory refers to the ability to actively retain, manipulate, and process information. During the first three years of life, basic aspects of executive functions (e.g., delayed response) emerge [3, 4]. Maturation towards more complex executive functions (e.g. cognitive flexibility,) occurs in a stepwise manner with one of the earliest key periods of rapid development occurring between the ages of three and five [3]. Despite this stepwise progression of skill executive function development, many studies have examined the most precise and reliable way to measure executive functions during this dynamic period, with most converging on the importance of using a single latent factors to best reflect children’s performance on multiple executive function tasks given their intersectionality in this early developmental period [5, 6].

Given the rapid growth of this developmental period, it is important to understand the contextual factors (e.g., household dynamic, parent–child interactions) that could influence the developmental trajectory of executive functions. Indeed, the underlying neural substrates (e.g. the prefrontal cortex) that subsume executive functions undergo prolonged development from childhood through to early adulthood [7, 8] making them vulnerable to the effects of early adversity [9]. As such, consideration of the quality of the home environment and parent–child interactions are important as they may shape the developmental trajectory of executive functions. Household chaos is one viable contextual factor that negatively impacts child executive functions [10]; however, mechanisms through which household chaos exerts its effects have yet to be fully elucidated. The current study explores whether household chaos influences child executive functions through parental responsiveness using a multi-method approach.

### Household chaos and child executive function

Household chaos has been widely conceptualized as an environment characterized by two dimensions: disorganization (e.g. clutter, ambient noise, crowding and lack of structure) and instability (e.g. frequent changes in residence, household occupants and routines) [11–13]. Most studies have assessed household chaos via parental report using the Confusion, Hubbub, and Order Scale (CHAOS) [14] and have demonstrated adverse effects on executive functions including inhibitory control, cognitive flexibility and working memory [15–17].

Given the broad characterization of household chaos, some studies have also examined the differential effects of the dimensions of household chaos on child executive functions. For example, greater household disorganization (e.g. household density, cleanliness), but not household instability, experienced in the first three years of life has been modestly, but significantly associated with lower performance in tasks measuring inhibitory control, attention shifting and working memory at age four [18]. Further, preschool children who were exposed to greater ambient noise demonstrated greater attention problems at kindergarten age; whereas a lack of routine was significantly associated with lower effortful control [19]. Similarly, children exposed to greater instability as measured by changes in caregivers, residences and parental employment, demonstrated greater difficulties with effortful control from ages four to six years [20]. Taken together, these studies suggest that it is important to consider both dimensions in evaluating the effects of household chaos on child executive functions.

### The role of parental responsiveness

Derived from attachment and sociocultural theoretical frameworks, parental responsiveness describes both an affective-emotional style (e.g., nurturing, contingent responses linked to child signals) and cognitively responsive behaviours (e.g., scaffolding, rich verbal input) [21]. These responsive interactions are essential to promoting optimal child outcomes, including child executive functions [22]. However, contextual risk factors such as household chaos, can compromise the quality of these responsive interactions [23]. Given these linkages, parental responsiveness may be a plausible factor through which household chaos exerts its effects on child executive functions, although this mechanism is rarely studied.

### Parental responsiveness and child executive functions

Early childhood is an essential time to establish a secure bond between parent and child, with long-term ramifications on child outcomes [24] including children’s executive functioning [22, 25, 26]. Early experiences with sensitive and responsive caregiving were positively associated with inhibitory control, working memory and cognitive flexibility in four and five year old children [27]; and, the effects of parenting are seen across development. A recent meta-analysis demonstrated that positive (e.g., sensitivity; *r* = 0.25), negative (e.g., intrusiveness; *r* = − 0.22) and cognitive (e.g. scaffolding; *r* = 0.20) parenting behaviours were significantly associated with children’s executive functions (up to age eight) [28]. Further, negative associations were demonstrated between inconsistent discipline and performance on tasks of inhibitory control, working memory and cognitive flexibility in adolescence (i.e., ages 9–14) [29]. Notably, parenting practices can also influence intergenerational transmission of risk. For example, positive parenting (e.g. physical and verbal affection) mitigated the negative effects of low socioeconomic status as experienced by the child’s grandparent, on the child’s inhibition and cognitive flexibility [30]. Taken together, these studies demonstrate that the quality of parent–child interactions plays an important, lasting role in shaping the development of executive functions in children.

### Household chaos and parental responsiveness

Several studies highlight the negative associations between household chaos and parenting [23, 31, 32]. A recent meta-analysis, for example, has demonstrated that chaotic homes are linked with increased conflict between parent and child, decreased supportive parenting, less effective disciplinary practices and greater hostility [33]. Chaotic home environments are also associated with less responsiveness, sensitivity and involvement of parents with their children [34]. Further, greater chaos and poor housing conditions are also associated with less warmth and greater negativity from parents as well as higher exposure to stressful events [35]. Taken together, this suggests that greater household chaos may be associated with a lower quality of parent–child interaction; thereby potentially exerting its effects on child executive functions via its influence on parental responsiveness.

### Household chaos, parental responsiveness and child executive functions

Various studies have demonstrated the mediating role that the quality of parent–child interactions play in the association between indicators of poverty and its related risk factors (such as household chaos) and child executive functioning. For example, higher cumulative risk (e.g., based on marital status, education training, number of children in household, household income, maternal age at birth) was found to be indirectly associated with lower executive functioning via early experiences of lower parental sensitivity for European American children and higher negative and intrusive parenting for African American children [36]. Similarly, indicators of poverty (e.g., income-to-need, maternal education) experienced in early childhood were associated with lower executive function performance via negative parenting (e.g., intrusiveness, negative regard) [37]. Finally, another study demonstrated that children exposed to various combinations of ecological risk (e.g., based on income, marital status etc.) demonstrate lower executive functioning performance at 36 months via lower maternal positive engagement and higher negative intrusiveness during infancy [38].

Although related to the construct of poverty [39, 40], studies have also demonstrated the specific indirect effects of household chaos on various child outcomes via parenting [41, 42]. Few studies, however, have examined parental responsiveness as a mediator between household chaos and child executive functions. For example, in a sample of school-aged children, parental positive reactions to child emotions significantly mediated the association between household chaos and child effortful control [43]. Additionally, cumulative household disorganization exerted a negative, indirect effect on the behavioural regulation of kindergarten-aged children via parenting acceptance and responsivity and early executive function skills [23]. The current study posits that parental responsiveness may be compromised under conditions of high household chaos which can adversely affect child executive functions. Using a kindergarten-aged sample, we extend the previous literature by implementing a multi-method approach to examine this mechanism; which involves the novel addition of narrated home video tours to assess the various aspects of household chaos including the absence or unpredictability of family routines.

### Current study

While evidence regarding the role of parenting in the association between household chaos and child executive functions is emerging, a few gaps remain. First, few studies have explored the potential differential effects of the dimensions of household chaos—disorganization and instability. The conceptualization of each dimension is broad; further, the frequency posed by elements of disorganization (e.g., ambient noise, clutter) may be greater than that of instability (e.g., changes in residences) whereas the reverse may be true regarding the level of risk posed. The empirical work to date is minimal, yet no studies, to our knowledge, have explicitly shown that each dimension has equal effect; therefore, further exploration is warranted. Second, the current study employs novel multi-method approaches in addition to self-report to provide a robust assessment of the associations between household chaos, parental responsiveness, and child executive functions. These included: 1) a standardized battery of performance-based executive function tasks for the mother and child; and 2) narrated home video tours (as a measure of household chaos) from which content in maternal speech is extracted. The use of linguistic analysis has shown that the words individuals use may reflect underlying psychological processes [44]; thus providing an innovative method to investigate household chaos that goes beyond the common practice of self-report.

Based on theoretical and empirical consideration, a number of covariates were included: sex of the child, household income, maternal executive functions, and depression. Child sex is a relevant covariate given demonstrated small developmental differences between male and female children in their executive functioning [45, 46]. While some studies have posited that the development of executive functions in male children may be slower than that of female children [47, 48]; potentially making them more vulnerable to contextual threats; others have not found significant sex differences in executive function developmental trajectories [49]. Thus, we controlled for child sex in our analyses. Household income, as a marker of socioeconomic status (SES), was also included as a covariate as low SES consistently predicts difficulties in child executive functioning [50]. Finally, maternal executive function and maternal depression were included as covariates given their respective associations with child executive functioning across various developmental time periods [51–53]. Studies have demonstrated a significant association between elevated maternal depression and greater chaos within homes [33].

The aims of the current study were to: (1) examine direct effects of household chaos on child executive functions and indirect effects via parental responsiveness; and (2) explore potential differential effects of instability and disorganization on parental responsiveness and child executive functions.

## Method

### Sample

A sample of 137 children and their mothers participated in the current study from June 2016 to August 2018. Most participants (*n* = 91) were a part of a larger longitudinal study examining the effects of maternal factors on parenting practices and child outcomes. Inclusion criteria were: (1) mothers were 18 years or older at time of birth; (2) mothers gave birth to full a term, healthy infant; (3) mothers were able to access their infants at the time of the home visits; and (4) mothers were able to read, write, and speak English. Exclusion criteria included any barriers to completion of research measures (e.g., severe disability, language barriers). For the current study, to bolster the sample size, additional participants were recruited using a database of families managed by the local university (*n* = 46). Eligibility of participants in the sub-sample matched that of the participants recruited from the longitudinal study. Families were contacted via telephone following a pre-approved script. Those who provided verbal, informed consent were scheduled for a home visit during which written, informed consent was obtained. The study protocol was approved by the local university and hospital ethics boards.

Nine mother–child dyads were excluded from data analysis. Two children had been diagnosed with severe developmental delay, and in seven cases, mothers had not completed the measures provided. The final sample consisted of 128 dyads (with 43 dyads coming from the supplemented sample). Mean child age was 61.9 months (*SD* = 2.0, range 58–68 months), with 49% females. Race composition consisted of 83.6% White, 2.3% Black, 2.3% Asian, and 11.7% Other (included those who reported more than one race). Most of the mothers were married or in common-law relationships (87.9%). Approximately 36.4% of mothers had university level training, 27.3% had a college education and 26.5% had post-graduate training. The remaining 9.8% had a high school diploma or less. The median household income ranged from $105,000 to 133,499 CAD. No significant differences were found between samples for SES or main variables with the exception of the Backward Digit Span (BDS) used as a measure of child working memory (*t*(122) = − 2.18, *p* < 0.05) where the supplemented sample had higher BDS scores than the original sample.

### Procedure

Two-hour home visits were conducted with mother–child dyads by two female research assistants. Mothers completed a questionnaire package addressing their emotional health and current stressors; their child’s health and development; and their household environment. The visit also included a video tour of the home conducted by mothers, videotaped mother–child interactions, and performance-based assessments of maternal and child executive functions. Participants were compensated $20 CAD for their time and were given a toy for the child.

## Measures

### Household chaos

#### CHAOS Scale

Mothers reported levels of household chaos with the Confusion, Hubbub, and Order Scale (CHAOS) [14]. The scale consists of fifteen items (e.g., “We can usually find things when we need them” and “Our home is a good place to relax.”) on a four-point scale (*1* = *very much like your own home, 4* = *not at all like your own home*). Higher scores indicate greater chaos in the home. The scale has high internal consistency in the present sample (Cronbach’s α = 0.85).

#### Family Inventory of Life Events and Changes (FILE)

Mothers completed the 9-item questionnaire assessing the family's experience of a variety of life events and changes [54]. For each event listed, mothers indicated whether they had experienced the event in the past month, past year, lifetime, never or prefer not to answer. The item used in the current analysis was ‘changes in relationship status (e.g., divorce, separation, remarriage, new partner)’ within the ‘past year’ due to its theoretical relevance to household chaos and in order to capture the relevant time period of the child’s life.

#### Residential mobility

As part of a demographic questionnaire, mothers indicated the number of times their child had moved from one residence to another in the past five years.

#### Narrated home video tours

Mothers conducted videotaped tours of their home based on a pre-established methodology [55]. Mothers were instructed to conduct a self-guided tour of their home during which they were to describe “*meaningful home spaces and possessions to their family*”. The research assistant did not accompany the mother on this tour. Most of the videos were continuous, however, there were few cases where videos were stopped and then resumed shortly after. Two independent researchers transcribed mothers’ dialogues verbatim, and a third researcher checked twenty percent of the videos. Where mothers engaged in extensive dialogue (i.e. beyond 2 lines of text) with other family members during the tour, the responses beyond the first reply were removed as recommended by Saxbe and Repetti [55]. Only the mother’s dialogue was transcribed. A Linguistic Inquiry and Word Count (LIWC) software was used to analyze transcripts of the home tours via generating word frequency counts for specified categories. To do this, a custom dictionary for household chaos was systematically created following a pre-established protocol of LIWC developers [44]. First, household chaos literature and a sub-set of the transcripts were reviewed to develop a codebook led by the first author. The codebook included broad categories of disorganization and instability. Sub-categories for ‘disorganization’ included words related to ambient noise, clutter, crowding, lack of structure; and sub-categories for ‘instability’ included words related to changes in caregivers, changes in residence, high home traffic patterns, lack of or unpredictable routines. Second, the codebook was entered into NVivo 12 Plus qualitative data analysis software [56] and used by the first author to extract and categorize chaos-relevant words and phrases from the transcripts. For example, words and phrases in the transcripts such as mess and noise were categorized under ‘disorganization’; whereas words and phrases such as routine and new residence were categorized under ‘instability’. The context within which the words and phrases were used were also considered to ensure accurate classification (e.g., “This is our messy playroom” would be categorized under disorganization; whereas “We’ve arranged the room this way so that it does not become messy” would not be categorized under disorganization). Third, the subsequent lists of words were reviewed and rated systematically by the first author and an independent trainee to create the final custom dictionary which was entered into the LIWC software to generate final word frequencies for the pre-determined household chaos categories. Reliability for the codebook and final dictionary were established via a similar multi-step process: (1) agreement from both reviewers was required for inclusion of words and phrases; and (2) discrepancies were resolved via discussion and consensus on inclusion or discarding.

### Parental responsiveness

#### Cognitive sensitivity and emotional availability

Mother–child dyads were videotaped as they participated in a structured interaction – the Etch-a-Sketch task [57]. Each were assigned a knob and instructed to not touch or manipulate the other’s knob as they completed a practice drawing (i.e., stacked rectangles), followed by the test drawing (i.e., a house). Parental responsiveness was coded using measures of cognitive sensitivity [58] and emotional availability [59]. Cognitive sensitivity includes indices for mind reading, communicative clarity, and mutuality-building behaviours [58]. Three coders (trained and reliable with the developer of the cognitive sensitivity scale (Cronbach’s α > 0.80)) used a 5-point Likert scale (range from 1 to 5) for each of the 11 items. A mean of the 11 items was used as the final score with higher scores indicating higher cognitive sensitivity. Emotional availability was measured using the 4^th^ edition of the Emotional Availability Scales (EAS) [59]. A primary coder (trained and EAS certified) scored the parent–child interaction videos using four parent scales—sensitivity, structuring, non-intrusiveness, and non-hostility (range from 1 to 7). A subset of 20 videos was coded by a second trained and certified EAS coder; reliability between coders was good (ICC = 0.81). Sum scores of the four scales was used and higher scores indicate higher emotional availability.

### Executive functions

Measures from the National Institute of Health (NIH) toolbox: Cognition Battery were used for mothers and children in assessing various executive functions. The NIH toolbox is a computerized battery of measures that can be administered to participants aged 3–85. Below, we provide a brief overview for each of the included NIH Toolbox tasks. See Weintraub [60] for more detailed descriptions.

#### Maternal executive functions

Mothers completed the Flanker Inhibitory Control and Attention Test (Flanker) and the Dimensional Change Card Sort (DCCS) from the NIH toolbox.

#### Flanker

In the Flanker task, a test of attention and inhibition, the mother was given up to three practice rounds with up to four trials each followed by 20 test trials. Scoring was based on both accuracy and reaction time and adjusted for age based on a normative sample. Higher scores indicate better attention and inhibitory control. Excellent reliability and validity has been demonstrated for this measure [60].

#### DCCS

For the DCCS task, a measure of cognitive flexibility, mothers completed two blocks: practice and mixed. In the practice block, mothers had to sort stimuli by dimension (i.e., shape or colour) and get at least three out of four correct in each round to move on. The mixed block included 30 test items and the dimensions, to sort by, alternated. The scoring for DCCS was the same as described for the Flanker test. Higher scores indicate better cognitive flexibility. Reliability and validity are excellent for this measure [60].

#### Child executive functions

Children also completed the Flanker and DCCS tasks as well as the Picture Sequence Memory Test (PSMT) as a part of the NIH toolbox. Children were also administered the Backward Digit Span (BDS) [61] and Simon Says [62] which were not a part of the NIH toolbox.

#### Flanker

The child version of the Flanker task was similar to the adult module; however, children were presented up to two test rounds. The first test trials consisted of 20 fish stimuli items. A score of greater or equal to 90% on these test trials was required for the child to move onto the additional 20 test trials with arrows as the visual stimuli. Both accuracy and reaction time were computed by the NIH toolbox. If the accuracy levels were below 80%, the computed score was equivalent to the accuracy score. However, if higher than 80%, reaction time was combined with the accuracy score. Higher scores indicate better attention and inhibitory control. Z scores were used in the current study for inclusion in a child executive function composite. Developmental sensitivity, test–retest reliability, and convergent validity of this task are all excellent for this age group [60, 63].

#### Dimensional change card sort

The child version of the DCCS consisted of four blocks: practice (same as adult module), pre-switch, post-switch and mixed. In the pre-switch block, the children matched the stimulus by colour for five test trials; with a score of four out of five correct needed to advance to the next block. In the post-switch block, the child sorted the stimulus by shape and again needed a score four out of five to move onto the mixed block. In the mixed block, the matching dimension of the stimulus alternated between colour and shape for 30 trials. Both accuracy and reaction time were computed by the NIH toolbox. If the accuracy levels were below 80%, the computed score was equivalent to the accuracy score. However, reaction time was combined with the accuracy score in the cases in which the level was 80% or higher. Higher DCCS scores indicate better cognitive flexibility. Z scores were used in the current study for inclusion in a child executive function composite. This measure has high reliability and convergent validity with developmental sensitivity throughout childhood [60, 63].

#### Picture sequence memory task

In the PSMT, a measure of episodic memory, children were administered two practice rounds with four trials each. If the child failed all four trials of a given practice round, the test was discontinued. Those who proceeded to the test round completed two trials of a nine-step sequence (i.e., how to play in the park). The test ended once the two trials were completed. A theta score (representing the overall performance) is generated from the number of adjacent pairs of pictures placed correctly in each of the two trials. The score is adjusted for age based on normative data. Higher scores indicate better episodic memory ability. Z scores were used in the current study for inclusion in a child executive function composite. This test has demonstrated high test–retest reliability and construct validity [64, 65].

#### Backward digit span

For the BDS, a test of working memory, children were instructed to repeat a sequence of numbers backwards. Three practice rounds with three trials each were provided with two digits. The test rounds (two items with two sets of numbers) began with two digits and increased in the number of digits in subsequent trials. The test was terminated when the child gave incorrect responses for both items in a trial. The total number of correct items was converted to a Z score for inclusion in a composite of child executive functions.

#### Simon says

In Simon Says, a measure of inhibition, children were instructed to follow the action of the research assistant only if the command was prefaced with ‘Simon says’ (directive trials); and not to follow if the command did not begin with ‘Simon says’ (inhibition trials). Following the practice rounds, ten test trials were conducted with five ‘directive’ trials and five ‘inhibition’ trials. Scores ranged from zero to three with a maximum score of 30. Higher scores indicate better inhibition. The total raw score was converted to a Z score for inclusion in a composite of child executive functions.

### Maternal depression

Mothers completed the 20-item Center for Epidemiologic Studies-Depression Scale (CES-D) [66] as a measure assessing depressive symptomology over the previous week. For each item, response options range from 0 (Rarely or None of the time) to 3 (Most or Almost All the Time). The clinical cut-off was a score of 16 or higher. Higher scores indicate higher levels of depressive symptomology. The CES-D has demonstrated high internal consistency and test-re-test reliability as well as adequate internal, concurrent and predictive validity with clinical ratings of depression and related self-report measures [66]. The measure for the present sample demonstrated good internal consistency (Cronbach’s *α* = 0.89). The total raw score was used in the current study.

### Statistical analyses

Descriptive statistics and bivariate correlations were calculated via SPSS v.26. All analyses were conducted using Mplus 8.3 [67]. Two composite variables were included in the analysis, household chaos and parental responsiveness. Variables were transformed into Z scores (with mean zero and standard deviation one) and summed [68]. For the total household chaos composite, variables were categorized into disorganization or instability informed by theoretical and empirical models and confirmed with principal component analysis (see Table 1). For the disorganization dimension, we combined the total score for the CHAOS Scale and the LIWC word frequency count referencing clutter, ambient noise, and lack of structure. Component weights were 0.65 and 0.82 for the CHAOS scale and LIWC word frequency count, respectively. For the instability dimension, we combined the FILE item, the residential mobility item, and the LIWC word frequency count referencing caregiver changes, residential changes, frequent visitors in and out of home and unpredictable routines; component weights were 0.72, 0.66, and 0.79, respectively. Both the ‘disorganization’ and ‘instability’ variables were combined into a composite to represent a ‘total household chaos’ construct. The parental responsiveness composite included mean scores for cognitive sensitivity and total scores for EAS which were highly correlated (*r* = 0.57, p < 0.01).

**Table 1:** Principle component analysis of the total household chaos composite

	Component	
	1	2
NHVT instability	0.79	
Changes in relationship status in past year	0.72	
Moves in past five years	0.66	
NHVT disorganization		0.82
CHAOS Scale total		0.65

*NHVT* narrated home video tour, *CHAOS* Confusion, Hubbub, and Order Scale

The measurement model for child executive functions (latent construct) was examined using confirmatory factor analysis (CFA) with Z scores of the indicators: Backward Digit Span, Simon Says and the NIH toolbox cognition composite (i.e., age-adjusted scores for Flanker test, DCCS, PSMT). The use of a latent variable model is aligned with literature suggesting greater reliability and precision of measurement [2].

Structural equation modeling was used to assess the direct effects of household chaos on child executive functions and the indirect effects via parental responsiveness (Model 1). Examination of the indirect effects followed the recommendation of using bias-corrected (BC) bootstrap confidence intervals (CIs) which accounts for non-normality of estimates and provides the greatest statistical power [69].

Covariates included were selected based on theoretical considerations and model parsimony: sex of child (coded as 1 = male, 0 = female), household income (combined maternal and paternal salaries), maternal executive functions (total scores for each of NIH Toolbox Flanker and DCCS) and maternal depression using total scores from the CES-D. A secondary analysis included examination of differential effects of each dimension of household chaos on child executive functions via parental responsiveness (disorganization and instability in Models 2a and b respectively). Measurement and structural models were assessed using the following indices of model fit: the likelihood ratio chi-square test, comparative fit index (CFI) and root mean squared error of approximation (RMSEA). As per standard recommendations, a non-significant chi-square test, and values of CFI greater than 0.95 and RMSEA less than 0.05 are considered indicative of good model fit [70].

### Missing data

Missing data ranged from 9.6 to 15.1% for main study variables. Full Information Maximum Likelihood Estimation (FIML) [67] within the structural equation modeling framework was used to account for missing data and non-normal distribution. This approach is considered to be superior to other traditional methods (i.e. listwise deletion, pairwise deletion, multiple imputation) [71] as it retains statistical power and produces unbiased estimates.

## Results

### Descriptive statistics

Means and standard deviations for main study variables as well as bivariate correlations are presented in Table 2. Each of the indicators of child executive functions were positively correlated with one another and with parental responsiveness. Disorganization and instability were not significantly correlated with either the indicators of child executive functions or parental responsiveness. The total household chaos composite, however, was significantly, negatively associated with parental responsiveness.

**Table 2:** Means, standard deviations and bivariate correlations for main study variables and covariates

Variables	Mean	SD	1	2	3	4	5	6	7	8	9	10	11
NIH toolbox composite (child)	104.10	15.05	1	.21*	.26**	− .11	.03	− .13	.27**	.06	.04	.25**	− .13
Backward Digit Span	3.62	1.76	.21*	1	.42**	− .14	− .05	− .06	.22**	.05	.12	.02	− .01
Simon Says	43.60	10.43	.26*	.42**	1	− .10	.001	− .17	.19*	.06	.06	.23*	− .04
Household chaos	0	.69	− .11	− .14	− .10	1	.74**	.50**	− .20*	− .05	.38**	− .16	− .02
Household disorganization	.01	.77	.03	− .05	.001	.74**	1	− .09	− .12	− .07	.34**	− .04	− .07
Household instability	0	.72	− .13	− .06	− .17	.50**	− .09	1	− .09	− .03	.09	− .08	.08
Parental responsiveness	0	.89	.27**	.22*	.19*	− .20*	− .12	− .09	1	.11	− .17	.14	.06
Maternal executive function	0	.88	.06	.05	.06	− .05	− .07	− .03	.11	1	− .17	.07	.10
Maternal depression	7.79	7.28	.04	.12	.06	.38**	.34**	.09	− .17	− .17	1	− .29**	− .08
Household income (median, CAD)	$105,000 – 133, 499		.25**	.02	.23*	− .16	− .04	.08	.14	.07	− .29**	1	− .12
Sex of Child (% female)	49		− .13	− .01	− .04	− .02	− .07	.08	.06	.10	− .08	− .12	1

^*^p < .05; ** *p* < .01

The dictionary and descriptive statistics for the home video tours are shown in Table 3. Tours ranged from 19 s to 14.85 min (*M* = 4.42 min, *SD* = 2.54) in length and the word count ranged from 49 to 1980 words (*M* = 521.81, *SD* = 377.12). Word frequency counts represent the number of words in a category as a percentage of the total number of words in a transcript. For example, a mean of 0.35 and 0.15 for disorganization and instability respectively, means that on average, for the mean tour length of approximately 522, 1.8 words per tour related to disorganization and 0.78 to instability. As such, the disorganization category was more frequently discussed in home tours with the most widely used words related to clutter and mess (75 tours). Each word in the dictionary was used at least once in two distinct home tours. The mean base rate for disorganization and instability was 26% meaning that approximately 33 of 128 tours included words from both categories.

**Table 3:** Percentage of words in home tour custom categories for household chaos construct

Category	Disorganization	Instability
Words included	chao*; clutter*; disarray; disast*; disorganiz*; hoard*; mess*; nois*; reno*; unclean; unfinished; untidy	attempt; entertaining; guests; new residence; novel; separation; visit*
Min to max	0–2.41	0–1.92
Mean	.35	.15
Median	.25	0
Standard deviation	.43	.30

Asterisks denote variants of the word that were also included in the Linguistic Inquiry and Word Count dictionary

### Household chaos, parental responsiveness and child executive functions

**Fig. 1:**
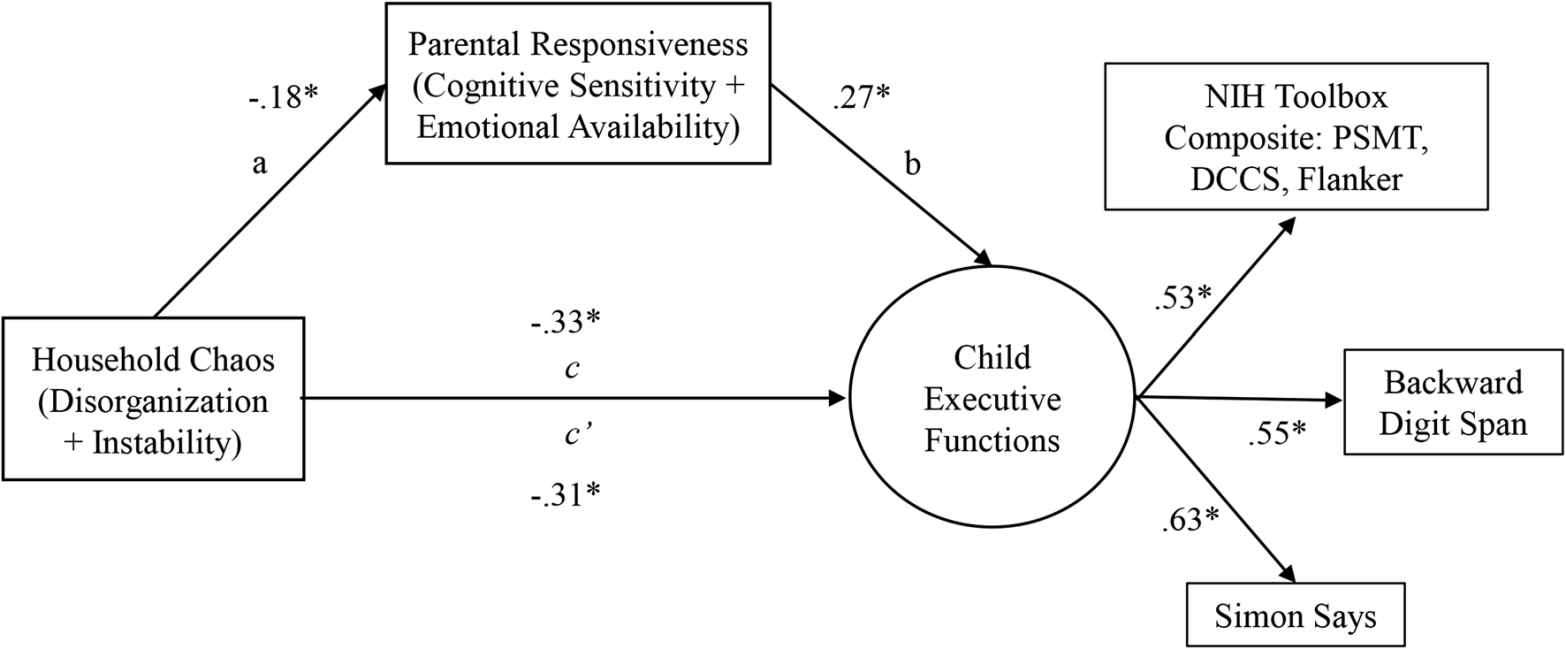
Structural equation model of direct and indirect effects of household chaos on child executive functions. *Note* Standardized loading coefficients shown. *PSMT* picture sequence memory test, *DCCS* dimensional change card sort, *Flanker* Flanker inhibitory control and attention test

The structural equation model examining the indirect path from the total household chaos composite to child executive functions via parental responsiveness (Model 1, Fig. 1) demonstrated excellent model fit (*n* = 128, χ^2^ (19) = 13.72, *p* = 0.80; RMSEA = 0.00; CFI = 1.00). The total effect from the total household chaos composite to child executive function was significant (path c: β = − 0.33, 95% CI [− 0.56, − 0.11]). Higher scores for the total household chaos composite were significantly associated with lower parental responsiveness. A significant positive association was also found between parental responsiveness and child executive functions. Finally, the indirect effect of the total household chaos composite on child executive functions via parental responsiveness was also significant (path ab: β = − 0.05, 95% CI [− 0.13, − 0.01]). Income was the only significant covariate in the model (β = 0.32, 95% CI [0.05, 0.56]). The model explained 28% of the variance of the latent construct of executive functions.

### Differential effects of household disorganization and instability

The differential effects of the dimensions of household chaos were assessed via secondary analyses (Models 2 a-b). Standardized coefficients of all paths are presented in Table 4. The model fit (Model 2a, disorganization) was excellent (*n* = 128, χ^2^ (19) = 14.84, *p* = 0.73; RMSEA = 0.00; CFI = 1.00). The total effect as not significant (path c: β = − 0.12, 95% CI [− 0.35, 0.11]). Nor were there significant direct or indirect effects of household disorganization on child executive functions. Income was the only significant covariate in the model (β = 0.31, 95% CI [0.03, 0.59]). Overall, the model explained 20% of the variance of the executive function construct.

**Table 4:** Structural model parameter estimates for household disorganization, household instability, parental responsiveness, and child executive functions (Model 2a and 2b)

	Standardized coefficient	95% CI
*Model 2a*		
Household disorganization → child executive functions (path c′)	− .12	[− .41, .16]
Household disorganization → parental responsiveness (path a)	− .08	[− .23, .07]
Parental responsiveness → child executive functions (path b)	**.29**	**[.08, .50]**
Household disorganization → parental responsiveness → child executive functions (path ab)	− .02	[− .09, .02]
*Model 2b*		
Household instability → child executive functions (path c′)	**−** **.29**	**[−** **.55, −** **.01]**
Household instability → parental responsiveness (path a)	**−** **.17**	**[−** **.33, −** **.002]**
Parental responsiveness → child executive functions (path b)	**.27**	**[.07, .46]**
Household instability → parental responsiveness → child executive functions (path ab)	**−** **.05**	**[−** **.12, −** **.004]**

Bolded effects are statistically significantly different from zero

For household instability (Model 2b), the model fit was excellent (*n* = 128, χ^2^ (19) = 15.85, *p* = 0.67; RMSEA = 0.00; CFI = 1.00). The total effect was negative and significant (path c: β = − 0.31, 95% CI [− 0.53, − 0.09]). Both direct and indirect effects were significant in this model. Income emerged as the only significant covariate in the model (β = 0.29, 95% CI [0.02, 0.55]). Overall, the model explained 28% of the variance of the executive function construct.

## Discussion

This study investigated parental responsiveness as a mechanism through which household chaos affects child executive functions. Using a novel, multi-method approach, we showed small but significant correlations between household chaos and child executive functioning such that greater household chaos was associated with lower performance on child executive function tasks, both directly and via lower parental responsiveness. Separating the dimensions of household chaos demonstrated that instability, and not disorganization, had a negative association with child executive functions, both directly and via parental responsiveness.

Greater chaos in the home has been previously linked to deficits in executive functions measured via direct assessments [10, 15] and questionnaires [17, 72]. Children may be withdrawing from uncontrollable environmental stimuli, in chaotic households [73], and while this may be protective against threats, it can constrain exposure to positive influences, thereby reducing opportunities for important interactions that promote executive function development. It is also possible that unpredictability instills a sense of helplessness as the child cannot control the events within their environment [74]. Thus, without the proper support (e.g. from a caregiver), this could hinder the child’s ability to manage their affect and behaviours, which have been linked to underlying deficits of executive functions [27].

### Indirect effects of household chaos

To our knowledge, few studies have formally examined the indirect effects of household chaos on child executive functions via parenting [23, 43]; making the findings of the current study particularly important to our understanding of this mechanism. The current findings are generally consistent with those of Vernon-Feagans et al. [23] wherein greater household chaos was associated with lower levels of positive parenting practices, which is related to poor executive functioning in their children. Chaotic homes may be linked to a parent’s struggle to regulate their own affect which can reduce their ability to engage in sensitive interactions with their child [31]. These compromised interactions can negatively affect the child’s executive function development [22]. Notably, given the cross-sectional design and the potential influence of other relevant factors not included in the current model, it would be important for future research to employ longitudinal designs with a more robust set of covariates in order to better elucidate the possible causal effect of household chaos on child executive functioning via parental responsiveness. Ultimately, understanding how chaotic homes influence the quality of parent–child interactions can inform programs aimed at supporting children and families.

### Household instability

Household instability emerged as a potentially important adverse predictor of child executive functions both directly and indirectly via parental responsiveness. The direct association between household instability and child executive functions is aligned with other studies [17, 20, 75]. However, examination of the indirect effect has been sparse. Extant literature has shown significant negative linkages between instability and maternal supportive parenting, which in turn, was negatively associated with child externalizing behaviour problems at kindergarten-age [76]. Perhaps, each transition forces the parent to adjust to a new setting, partner (or loss of one) and/or routine [77]. If the parent is unable to adjust to such transitions, possibly due to depleted energy, increased frustrations or preoccupations [78], this may negatively impact their interactions with their child. Interestingly, only one study to date has examined the differential effects of household chaos dimensions in a similar mediation model; however they found that household disorganization, and not instability, indirectly predicted child regulatory behaviours via parenting [23]. It is possible that since instability was measured at an earlier age (i.e. 2–36 months), transitions may not have been as disruptive as compared to the current sample of school-aged children who are beginning to establish ties to their community, peers and school [12]. Also, while the current study included measures of routines, Vernon-Feagans et al. [23] did not. Perhaps, the absence or unpredictability of routines has a unique, salient adverse effect on the executive functions of the children. This is supported by empirical evidence of the importance of routines in the development of regulatory processes [19, 79]. With this said, given the combination of the small but significant correlation provided in the current study and the cross-sectional design limiting any causal inferences, further studies are needed to replicate these findings and elucidate the potential differential effects of the elements of instability.

### Income

Notably, income was the only covariate that consistently emerged as significant in all of the primary and secondary analyses. However, the significant effect of household chaos on parenting and child executive function was sustained with the inclusion of income, further supporting the notion that household chaos is not simply a proxy for income [32, 72]. Future research should consider a more comprehensive investigation into the possible differential effects of household chaos across SES gradients via an income-stratified analysis with a larger, representative sample.

### Strengths and limitations

The current study expands the growing literature examining the associations between household chaos, parental responsiveness and child executive functions via a novel, multi-method approach. For example, the home tours completed by mothers can provide spontaneous and automatic responses, adding to the ecological validity of the household chaos construct as opposed to the controlled responses one obtains via structured, standardized questionnaires. Further, our use of video-taped interactions to assess parental responsiveness and a battery of performance-based executive function tasks mitigates possible shared method variance, likely an issue with previous studies largely reliant on report measures. Further, maternal characteristics that could be affected by household chaos and impact mother–child interactions were controlled for, including maternal executive functions [80] and mood [32]; previous studies typically focus on SES factors only.

Despite the study strengths, it is not without limitations. The cross-sectional design prevents assessment of the effects of household chaos over time. Although we showed that even small doses of instability are linked to parental responsiveness, assessing cumulative effects on parenting and child executive functions over time should be a future research direction. It would provide greater insight into the directionality of effect where possible reciprocal associations between household chaos and parenting and parenting and child executive functions could be thoroughly assessed. Additionally, given that executive function changes over time [81, 82], future studies should consider the differential effects of household chaos and parental responsiveness of child executive functions based on the age of the child. This would highlight developmental periods which may be more vulnerable to these contextual factors. Also, while the indirect effect of household instability on child executive functions via parental responsiveness was significant, its small effect suggests that more studies are needed to better elucidate the differential effects of household chaos dimensions on child executive functions. Additionally, despite the novelty of the narrated video home tours in measuring elements of household chaos, there was significant variability in the length of and verbosity within the home tours that should be controlled for in future research via standard protocols. Also, while a number of relevant covariates were included in the model, there are other factors (e.g., attachment quality, marital conflict), that could also affect household chaos, parental responsiveness and child executive functioning that were not included in the model. It is also important to note that only two measures of executive functions were used for mothers (inhibition and cognitive flexibility), however, future research should consider a more robust measurement battery including basic (e.g., working memory) and more complex (e.g., planning, problem solving) executive functions; this would give better insight into the potential influence of maternal executive functions on the study variables. Finally, as this was a low risk, ethnically homogenous sample, generalizability of the results may be limited. Future research is needed to replicate these findings in a larger, representative sample.

## Conclusion

This study provides a novel, multi-method approach to examine associations between household chaos, parental responsiveness, and child executive functions. It highlights parental responsiveness as an important factor partially explaining the association between household chaos and child executive functions. It also brings attention to the importance of structure, predictability, and routines within the home on the development of child executive functions. With additional support through replication and causally sensitive designs, both parenting and stability within the home are potential targets for interventions that could act to promote healthier developmental trajectories of executive functions in children.

## Data Availability

Upon publication of the results, the datasets generated and/or analysed during the current study will be available from the corresponding author on reasonable request.
